# Active Participatory Surveillance for Early Detection of Notifiable Pathogens: A Case Study of the U.S. Swine Industry

**DOI:** 10.3390/v18040478

**Published:** 2026-04-20

**Authors:** Berenice Munguía-Ramírez, Giovani Trevisan, Paul Morris, Gustavo S. Silva, Danyang Zhang, Chong Wang, Rodger Main, Jeffrey Zimmerman

**Affiliations:** 1 Department of Veterinary Diagnostic and Production Animal Medicine, College of Veterinary Medicine, Iowa State University, 1800 Christensen Drive, Ames, IA 50011-1134, USA; trevisan@iastate.edu (G.T.); gustavos@iastate.edu (G.S.S.); chwang@iastate.edu (C.W.); rmain@iastate.edu (R.M.); jjzimm@iastate.edu (J.Z.); 2 Department of Statistics, College of Liberal Arts and Sciences, Iowa State University, 2438 Osborn Drive, Ames, IA 50011-1090, USA; psmorris@iastate.edu (P.M.); zhangdy@iastate.edu (D.Z.)

**Keywords:** notifiable pathogens, participatory surveillance, swine diseases

## Abstract

The continued global spread of WOAH-listed pathogens via trade, transport, and travel calls for the implementation of biosecurity measures to protect the health of our national livestock industries, plus ongoing surveillance to verify that such measures are operative. Despite this urgency, surveillance must be practical and affordable. Herein, we evaluated the performance and cost of participatory surveillance, a nontraditional surveillance design, using the U.S. swine industry as an example. In this context, “participatory” meant that herd veterinarians and/or producers collected and submitted samples from the herd to accredited laboratories for testing. To create an infected population (Phase 1), we simulated the introduction and spread of an unspecified notifiable pathogen within the 48 contiguous U.S states (66,637 swine farms, within 8,080,470 km^2^) using the USDA Animal Disease Spread Model software (v3.5.10.0). In Phase 2, we calculated the probability of detecting ≥1 infected farm as a function of producer participation, farm-level sensitivity, farm-level prevalence, and sampling frequency. The participatory design was effective: ≥90% probability of detecting the notifiable pathogen at 0.05% farm prevalence (33 positive farms among 66,637 farms) when farm-level sensitivity was ≥20% and producer participation was ≥40%. Depending on the specimen collected, the shipment method, and the test selected, costs ranged from $0.03 to $0.07 USD (€0.02 to €0.06) per pig in inventory. Thus, a surveillance design based on collecting and testing specimens from a few targeted pigs on each of many farms would be both affordable and effective at a national level.

## 1. Introduction

Notifiable pathogens, i.e., diseases listed in the Terrestrial Animal Health Code (WOAH, 2024), can have substantial economic consequences if introduced into free areas. In Brazil, the control and eradication of African swine fever (ASFV) (1978–1986) cost $20,913,206 [1]. In Taiwan, the introduction of foot-and-mouth disease (FMDV) (1997) resulted in losses of $378.9 million [2]. In the Netherlands, the control and elimination of classical swine fever virus (CSFV) (1997) cost $2.3 billion [3]. In the United Kingdom, FMDV (2001) produced losses estimated at $12–18 billion [4]. In the United States (U.S.), the emergence of porcine epidemic diarrhea virus (PEDV) (2013) cost pork producers ~$1 billion, primarily due to the loss of export markets [5,6,7].

The ongoing international spread of notifiable livestock pathogens is facilitated by global trade in live animals, meat, and meat by-products [8,9,10,11,12,13]. In 2022, international trading networks transported 41.8 million tonnes of meat and meat products, 11.5 million tonnes of which constituted pig meat. In addition, ~73 million live animals (sheep, goats, cattle, and pigs) were traded between countries, including 47 million live pigs [14,15]. These numbers reflect a vibrant international agricultural economy but also a significant “opportunity” for pathogens of consequence to move between countries.

In addition to trade, human travel likewise plays an important role in the transboundary movement of pathogens. For example, the outbreaks of FMDV in Canada (1951), ASFV in Brazil (1978), ASFV in Sweden (2023), and FMDV in Germany (2025) are all believed to have been caused by people carrying contaminated fomites into disease-free countries [1,16,17,18].

If prevention is better than cure, it follows that each country needs to prevent the introduction of notifiable pathogens into its livestock industries via enhanced biosecurity, immunization, and other proactive measures. Underlying these efforts, ongoing surveillance is needed to verify that the measures put in place are functioning correctly. The dilemma is that the cost of surveillance can be burdensome. Benedictus et al. (2009) estimated the cost of preventing bovine spongiform encephalopathy from entering the human food chain in the Netherlands at ~2 million euros per life-year saved [19]. Similarly, Drewe et al. (2014) reported that Great Britain spent ~47.3 million pounds on livestock health surveillance in 2011 [20]. Few countries have the fiscal resources and political will required to sustain this level of commitment over time.

There is no universally accepted national surveillance strategy [21], and most countries employ both syndromic and risk-based schemes. An under-used alternative is participatory surveillance, which relies on the collaboration of the members of the community at risk. For example, the global smallpox eradication program (1967–1977) provided a bounty to members of the community for identifying smallpox cases [22]. This approach resulted in the identification of the world’s last naturally acquired smallpox case in 1977 [23]. In the 1990s, for participatory surveillance of human populations, parties began to use the internet and social media to collect data [24]. For example, the “de Grote Griepmeting” (Great Influenza Survey) launched during the 2003 influenza season collected data on influenza infections from Dutch-speaking participants via the website “Influenzanet” [25]. Subsequent analyses of Influenzanet data have revealed associations between risk factors and influenza symptomatology and helped to refine preventive measures, e.g., vaccination campaigns [26].

In veterinary medicine, because the internet is not necessarily accessible in rural areas, participatory surveillance has relied on face-to-face interviews to actively collect clinical observations and epidemiological information, with diagnostic follow-ups used to establish true infection status [27,28]. This design was used in rinderpest surveillance in eastern Africa (1994–2001) [29] and highly pathogenic avian influenza virus (H5N1) surveillance in Egypt (2008) [27,30]. However, the participatory approach actually began much earlier in veterinary medicine [31,32]. For example, the U.S. poultry industry established the National Poultry Improvement Plan (NPIP) in 1935 as a voluntary, participatory, and collaborative effort among producers, industry, and government. Initially organized to control pullorum disease, the NPIP thereafter evolved in response to poultry disease challenges, e.g., *M. gallisepticum*, *M. synoviae*, *Salmonella pullorum*, and avian influenza virus (AIV) [33]. Within the NPIP, certification of flock status is based on the ongoing collection and testing of samples by accredited licensed flock veterinarians or certified technicians [33,34]. Thus, the NPIP AIV H5/H7 Monitored certification of commercial poultry (9 CFR § 146) collects serum or egg samples of ≥11 birds at 21-day intervals with approved testing for H5/H7 AIV antibody, nucleic acid, or antigen performed at authorized laboratories.

At present, the U.S. is the world’s third-largest producer and consumer of pork and pork products, accounting for approximately 11% of global production in the 2023/2024 marketing year, with a total production of 12.39 million metric tons [35]. Following the NPIP example, U.S. swine producers, industry, and government are working to develop the U.S. Swine Health Improvement Plan (U.S. SHIP) [36,37]. U.S. SHIP’s initial focus centers on establishing an ASFV/CSFV Monitored certification, modeled after the NPIP H5/H7 Monitored certification held by U.S. Commercial Poultry operations. In the event of the introduction of ASFV or CSFV into the U.S., for example, participant swine producers would submit ≤10 diagnostic samples (depending on farm type and specimen) from animals in poor health on a monthly basis for testing in accredited laboratories. The underlying assumption of this approach is that a few samples from many farms could effectively achieve regional detection of a targeted pathogen. In a pilot study, Trevisan et al. (2024) reported that this design was highly effective, e.g., achieving a 97% probability of detecting ≥1 positive farms at 0.1% prevalence (18 farms) in a region containing 17,521 farms, given a farm-level sensitivity of 30% and producer participation equal to 60% [38]. The objective of the present study was to evaluate this approach across the continental U.S., i.e., 66,637 swine farms holding 71,555,218 pigs across an area of 8,080,470 km^2^. In particular, our aim was to calculate the probability of early detection of a notifiable swine pathogen as a function of producer participation, farm-level prevalence, farm-level detection sensitivity, and sampling frequency. Furthermore, the costs of sample collection, submission, and testing at certified laboratories were calculated under the assumption that producers would cover these costs, as is the case for the NPIP.

## 2. Materials and Methods

This study was conducted in a stepwise fashion. Phase 1 simulated the introduction and spread of an unspecified notifiable pathogen among swine farms in the 48 contiguous U.S. states over a period of 180 days using the United States Department of Agriculture (USDA) Animal Disease Spread Model (ADSM) software (v3.5.10.0) [39]. ADSM is public domain software designed for the stochastic simulation of the spread of an infectious agent among a defined population of farms. Previously, it was used to simulate the spread of ASFV in Vietnam [40] and China [41], CSFV in the Republic of Serbia [42], and FMDV in Denmark [43]. In the present study, pathogen transmission was assumed to occur exclusively between swine, with no involvement of other species or wildlife.

In this study, rather than focusing on a specific pathogen, we used a range of transmission parameter values in the ADSM software to produce transmission outcomes generalizable to a variety of pathogens in the defined population of swine farms (Section 2.1.1). Each simulation was initiated on a randomly selected index farm, with pathogen transmission dependent on scenario-specific probability parameters associated with the movement of animals, personnel, and fomites within the region, as well as local-area spread between farms (Section 2.1.4). Because ADSM simulations are stochastic, 1000 iterations were performed for each disease-spread scenario (*n* = 6075). The output for each simulated scenario, i.e., the infection status of each farm according to the day post-introduction (DPI), provided the data for Phase 2 (probability of pathogen detection). Phase 2 simulated disease detection within the U.S. via the time post-outbreak as a function of farm-level prevalence, the proportion of farms participating in active surveillance, sampling frequency, and farm-level diagnostic sensitivity [44]. An R function (rbinom) was used to perform the disease detection (surveillance) simulations for each combination of spread scenario and detection settings. Phase 3 calculated the cost of active participatory surveillance for U.S. swine producers for surveillance-sampling scenarios (*n* = 8) based on different combinations of the specimens collected, shipping costs, and laboratory assays utilized.

### 2.1. Phase 1: Simulation of Pathogen Spread Among U.S. Swine Farms

#### 2.1.1. Dataset of U.S. Swine Farms Used in ADSM Simulations

Simulations were based on a swine farm dataset derived from the 2017 U.S. Census of Agriculture [45]. The U.S. Census of Agriculture is a nationwide survey of U.S. agriculture conducted by the USDA National Agricultural Statistics Service every five years, i.e., in years ending in “2” and “7”. As established in the 1974 Census, a “farm” is defined as “any place from which $1000 or more of agricultural products were produced and sold, or normally would have been sold, during the census year” [45]. After data had been curated and the focus was refined to solely the contiguous 48 U.S. states, the dataset contained 66,637 swine farms holding 71,555,218 pigs in a geographic area of 8,080,470 km^2^. In addition, 74 swine slaughter packing plants and their daily harvest capacities were included in the dataset to account for indirect pathogen transmission resulting from contamination of transport, i.e., trucks and trailers, at abattoirs [46,47].

#### 2.1.2. Adaptation of the U.S. Census of Agriculture for Use in ADSM

ADSM software required each farm to have a production type, precise inventory (number of pigs), and geolocation (longitude and latitude) identities. Because the Census did not provide this level of detail for all farms, the following procedures were applied.

**Production type:** To meet the ADSM requirement for production-type identification and conform to contemporary U.S swine industry classifications, Census production type categories (farrow-to-feeder, farrow-to-finish, farrow-to-wean, finish only, nursery, or “other practices”) were subsumed into one of three categories: breeder (farrow-to-feeder and farrow-to-wean), breeder–feeder (farrow-to-finish), and feeder (finish only and nursery) [37]. Using this approach, 56,228 of 66,637 farms in the ADSM dataset were identified by production type: 12,572 breeder, 20,835 breeder–feeder, and 22,821 feeder farms. The remaining 10,409 farms, including those with production types listed as “other practices,” were randomly assigned to a production type in proportion to state-level production types or, if state data were not adequately reported, the overall proportions in the ADSM dataset, using R v.4.1.0 [48].

**Farm inventory:** For all 48 contiguous states and their counties, the U.S. Census reported the total number of farms by inventory category (1 to 24, 25 to 49, 50 to 99, 100 to 199, 200 to 499, 500 to 999, 1000 to 1999, 2000 to 4999, and ≥5000 pigs). Of the 48 states, 45 also reported total state pig inventories, thereby accounting for 98.6% of all farms with swine in the U.S. However, ADSM software required a specific inventory for each farm, not just an inventory category. Therefore, when the Census provided the total number of farms and total inventory for a given state, the total inventory was divided among the farms as evenly as possible using R v.4.1.0 [rep(floor(total state inventory/# of farms), # of farms)], where “# of farms” refers to the total number of farms within the state belonging to a given production type and inventory category. In order to account for the total pig inventory, any remaining state-level inventory was distributed by adding one additional pig to randomly selected farms. For three states with low pig numbers, i.e., Arizona, Massachusetts, and Nevada, the Census did not report state-level total pig inventory, but it did report the number of farms by inventory category. Therefore, a farm-specific inventory was obtained through R v.4.1.0: sum(runif(*n* = # of farms, a = minimum inventory, b = maximum inventory)), where “minimum inventory” is the lower bound of the inventory category, and “maximum inventory” is the upper bound of the inventory category.

**Geolocation:** To protect producer confidentiality, the Census did not report farm geolocation. Therefore, for a given state and inventory category, farms were randomly assigned to counties according to the number of farms in each county. Thereafter, with the year argument set to 2017, the counties function from the tigris R package (v.1.6) was used to obtain shapefiles for each county in the contiguous U.S., and the st_sample function from the sf R package (v.0.9-7) was used to randomly generate latitude and longitude coordinates for each farm within the assigned county.

#### 2.1.3. Spatial Tessellation of the Geographic Area

**Figure 1:**
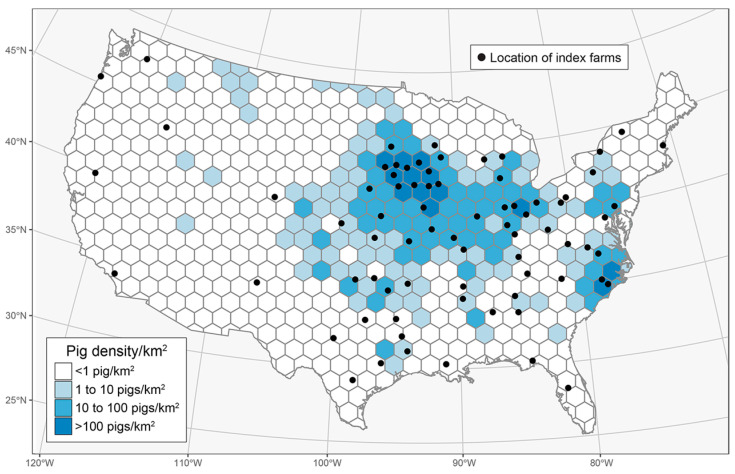
Index herd (*n* = 75) locations per pig density per km^2^ category in the contiguous U.S. region subdivided into 597 hexagonal geographical regions measuring 16,000 km^2^ (R v.4.1.0): <1 pig per km^2^ (30 index herds), 1 to 10 pigs per km^2^ (15 index herds), 10 to 100 pigs per km^2^ (15 index herds), and >100 pigs per km^2^ (15 index herds). Final density was calculated via the ratio of the pig inventory in the region to the area in km^2^. Using this approach, we found that 19 regions had zero pig inventories.

To better describe and analyze the spatial aspects of pathogen spread, the geographic area within the contiguous 48 states was divided into uniform hexagons (tessellation) using R v4.1.0 [49,50]. As shown in Figure 1, this resulted in 597 hexagons with ~78.5 km (48.8 miles) per side, each enclosing 16,000 km^2^ (6177 mi^2^). Pig density per km^2^ was calculated for each hexagon based on the 2017 U.S. Census of Agriculture [45], with partial hexagons, i.e., hexagons adjacent to borders, adjusted for their actual area. Through this approach, four pig density categories were identified: <1 pig per km^2^, 1 to 10 pigs per km^2^, 10 to 100 pigs per km^2^, and >100 pigs per km^2^.

#### 2.1.4. Disease Spread Simulations

To obtain disease spread results generalizable across a variety of swine pathogens, all combinations (*n* = 27) of the transmission probabilities listed in Table 1 for direct contact, indirect contact, and area spread were used in ADSM-simulated 180-day disease outbreaks. Notably, animal movement parameters used in the simulations reflected both intrastate and interstate pig movement. Interstate movement reflected the common industry practice of moving growing pigs from sow farms located at the periphery to farms located in the Corn Belt States, i.e., South Dakota, Nebraska, Minnesota, Iowa, Wisconsin, Illinois, Indiana, and Ohio [51,52]. Although disease control options, i.e., quarantine, destruction, and vaccination, were available in ADSM, they were not implemented to allow uncontrolled spread.

Each ADSM simulation was initiated on one randomly selected index farm. Taking all scenarios into account, a total of 75 index farms were selected based on pig density category, i.e., 30 index farms from the “<1 pig per km^2^” category and 15 index farms each of the “1 to 10”, “10 to 100”, and “>100” pigs per km^2^ categories. More index farms in the “<1 pig per km^2^” category were selected because of the assumption that disease spread would be constrained in less “pig-dense” areas. To account for the effect of production type on disease transmission, each index farm (*n* = 75) was assigned to each production type (breeder, feeder, and breeder/feeder) in separate simulations. Thus, a total of 6075 disease spread scenarios were simulated, that is, all possible combinations of index farm within pig density category (*n =* 75), index farm production type (*n* = 3), transmission by direct contact (*n* = 3), transmission by indirect contact (*n* = 3), and local area spread (*n* = 3). Each scenario was simulated 1000 times to account for stochasticity in the ADSM software. The combination of the ADSM and R software (v.4.1.0) allowed for the automation of the disease-spread process through the dbConnect function from the RSQLite R package (version 2.2.4) for integration with the SQLite database, as previously described [38].

### 2.2. Phase 1 Results: Pathogen Spread

**Figure 2:**
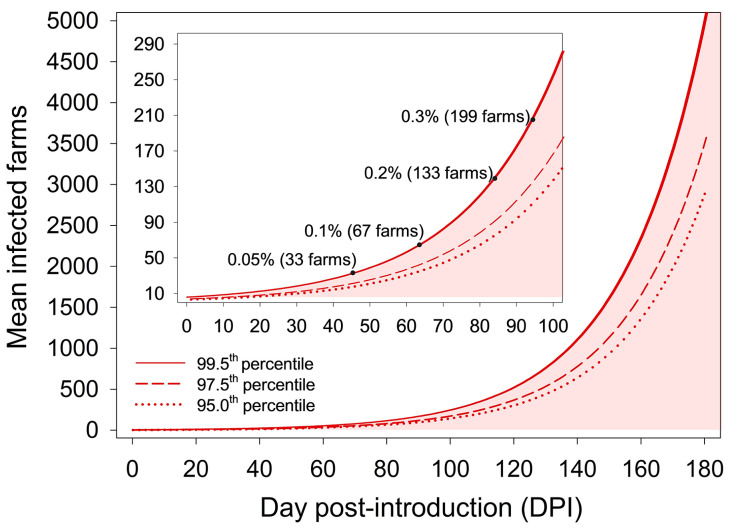
Mean infected farms by day post-introduction (DPI) resulting from 6075 disease spread scenarios (with 1000 iterations each). In the aggregate, ADSM simulation output at the 99.5th (solid line), 97.5th (dashed line), and 95th (dotted line) percentiles corresponded to 5222, 3574, and 2944 mean infected farms at DPI 180, respectively. The inset graph shows disease prevalence (0.05%, 0.1%, 0.2%, and 0.3%) according to DPI.

The ADSM output for the 6075 disease spread scenarios (1000 simulations for each scenario) is presented as the mean number of infected farms stratified by DPI in Figure 2. The ADSM output at the 99.5th, 97.5th, and 95th percentiles corresponded to 5222, 3574, and 2944 mean infected farms at DPI 180, respectively.

**Figure 3:**
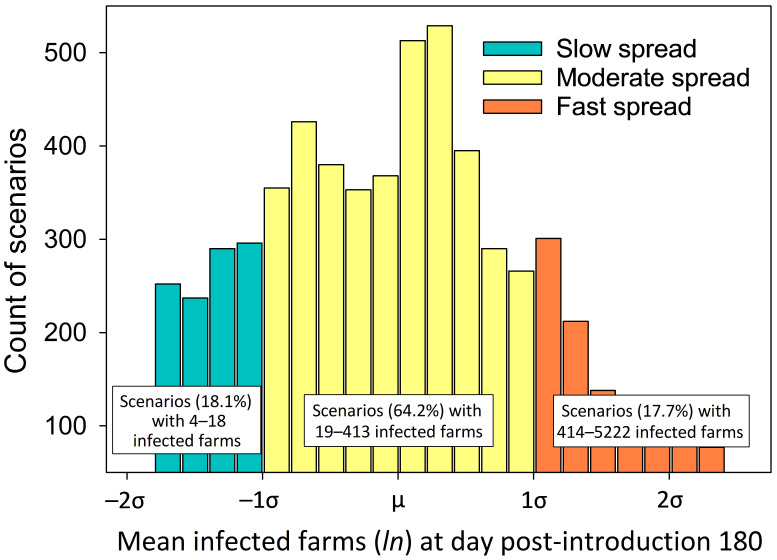
Aggregated log-transformed (*ln* x) mean infected farms at DPI 180 from ADSM disease spread simulations (6075 scenarios—mean is based on 1000 iterations per scenario). Moderate spread (μ ± 1σ; 3901 scenarios) resulted in 19 to 413 mean infected farms. Slow spread (−1σ to −2σ; 1100 scenarios) resulted in 4 to 18 mean infected farms. Fast spread (1σ to 2σ; 1074 scenarios) resulted in 414 to 5222 mean infected farms.

To identify the parameters driving differences in disease spread, 3 disease spread categories were defined based on the distribution of the log-transformed (*ln* x) mean infected farms at DPI 180 (Figure 3): moderate spread (μ ± 1σ) resulted in 19 to 413 mean infected farms, slow spread (−1σ to −2σ) resulted in 4 to 18 mean infected farms, and fast spread (1σ to 2σ) resulted in 414 to 5222 mean infected farms. These represented 64.2%, 18.1%, and 17.7%, respectively, of the total scenarios.

Linear regression (R v.4.3.3) analysis showed that direct contact, local area spread, pig density/km^2^, and production type significantly affected the number of infected farms at DPI 180 (*p* < 0.01), but indirect contact did not (*p* > 0.01). As shown in Table 2, direct contact probabilities of 0.2, 0.4, and 0.6 and local area spread probabilities of 0.001, 0.01, and 0.1 were associated with slow, moderate, and fast spread, respectively (*p* < 0.01; chi-squared (X^2^) test). Production type and pig density parameters were only significant for slow and fast spread categories. Slow spread was associated with breeder–feeder or feeder (*p* < 0.01; X^2^) index farms located in areas with <1 pig per km^2^ (*p* < 0.01; X^2^), and fast spread was associated with index farms in breeding herds located in areas with >100 pigs per km^2^ (*p* < 0.01; X^2^). This pattern likely reflects the higher frequency of pigs moving from breeder farms to feeder farms, while feeder farms generally only move pigs to slaughterhouses.

### 2.3. Phase 2: Simulation of Pathogen Detection Among Swine Farms in the U.S.

Among the 6075 disease spread scenarios simulated in Phase 1, a subset of 2025 scenarios (75 index farms × 3 production types × 3 direct contact transmission probabilities × 3 indirect contact transmission probabilities × a local area spread probability of 0.01) was used in Phase 2. Of these, 475 (23.4%) scenarios corresponded to slow spread, 1403 (69.3%) corresponded to moderate spread, and 147 (7.3%) corresponded to fast spread.

As described previously [38], the detection of ≥ 1 positive farm was simulated in R for each replicate (*n* = 1000) of the 2025 spread scenarios for each of the 25 pathogen detection settings, i.e., 5 farm-level sensitivity levels (10%, 20%, 30%, 40%, and 50%) and 5 producer participation levels (20%, 40%, 60%, 80%, and 100%). For each producer participation level, participation was allocated uniformly across the four pig density categories (<1 pig per km^2^, 1 to 10 pigs per km^2^, 10 to 100 pigs per km^2^, and >100 pigs per km^2^) through simple random sampling without replacement. For example, simulations at 20% producer participation included 20% of the farms in the “<1 pig per km^2^” category, 20% of the farms in the “1 to 10 pigs per km^2^” category, and so on for the remaining two categories. By definition, 100% participation did not require participant selection.

The probability of detection, defined as the percentage of iterations in which ≥1 true-positive farm was identified, was calculated in two ways (R v.4.3.3): as a function of farm-level prevalence and as a function of time (DPI). Farm-level prevalence represented the proportion of farms infected at a given time point under the assumption that once a farm was infected, it remained infected for the duration of the simulation.

The probability of detection at specific farm-level prevalences (0.01%, 0.05%, 0.10%, and 0.20%) was calculated as the weighted average probability of all simulation results matching the selected prevalences (7, 33, 68, and 133 infected farms, respectively) for each combination of producer participation and farm-level sensitivity.

The probability of detection according to DPI was compared between single point-in-time sampling and the aggregate detection probability based on repeated sampling (14-day intervals) for all levels of producer participation and farm-level sensitivity. The probability of detecting ≥ 1 positive farm during a single sampling instance (*P*) was calculated as the complement of the probability of detecting zero positive farms among participating farms (Equation (1)) at a specific DPI. In Equation (1), “*p*” represents the probability of not detecting any positive farms (the probability of failure), and “*n*” is the percentage (%) of positive participating farms [55].

*P* = 1 − (1 − *p*)*^n^* (1)

The aggregated probability of detecting ≥1 positive farm with repeated sampling (*Pa*) was calculated as the complement of the combined probability of zero detection across all sampling time points (Equation (2)). In Equation (2), “*P*_14_, *P*_28_…*P_DPI_*” denotes the individual probabilities of detecting at least one positive farm at a specific DPI.

*Pa =* [1 − (1 − *P*_14_) × (1 − *P*_28_) ×… (1 − *P*_DPI_)] (2)

#### Phase 2 Results: Probability of Pathogen Detection in Swine Farms in the U.S.

Table 3 provides detection probabilities at specific farm-level prevalences over a range of producer participation and farm-level sensitivity levels. Overall, a producer participation of ≥40% with a farm-level sensitivity of ≥20% consistently produced a ≥90% probability of detection given a farm prevalence ≥ 0.05% (≥33 farms). A linear regression model showed that the detection probability significantly increased at higher levels of producer participation (*p* < 0.01) and farm-level sensitivity (*p* < 0.01), but no interaction was detected between producer participation and farm-level sensitivity (*p* > 0.05).

Table 4 provides a comparison of the probability of detection over time post-introduction for single-point-in-time sampling vs. repeated sampling (14-day intervals). Notably, repeated sampling consistently improved detection in all cases where point-in-time sampling provided a probability of detection less than 100%.

### 2.4. Phase 3: Cost of Sampling and Testing

**Figure 4:**
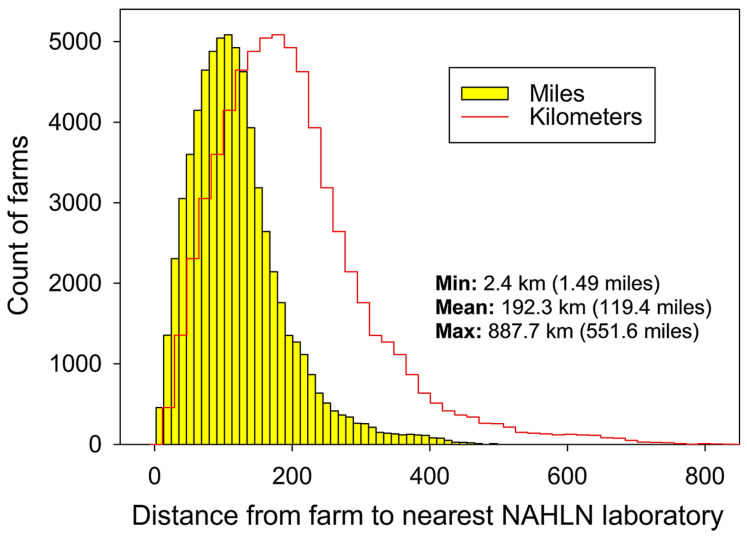
Distance from swine farms (*n* = 66,637) used in Phase 1 simulations to the nearest National Animal Health Laboratory Network (NAHLN) laboratory, calculated as the shortest driving distance using data from OpenStreetMap and the open-source routing machine method (ORSM) [59].

National active participatory surveillance sampling and testing costs were calculated under the following assumptions:

1. On-farm sampling would be performed under the supervision of a USDA Category II accredited veterinarian using a targeted sampling approach [56,57]. The labor required for collecting and processing samples on farms would be provided by producers free of charge for the program. This assumption reflects current practices in existing voluntary programs such as the NPIP, where producer labor for routine surveillance activities is not monetized separately [33].

2. Swine farms with >2000 pigs in inventory would collect 10 oral swab or blood swab samples from individual poor-doing pigs or 2 oral fluid samples from pens holding poor-doing pigs. Farms with ≤2000 pigs would collect 5 oral swabs or blood swabs from individual poor-doing pigs or 1 oral fluid sample from a pen holding poor-doing pigs. Individual swab samples would be tested for nucleic acid (PCR) in pools of five; individual oral fluids would be tested for nucleic acid (PCR) or antibodies (ELISA). Thus, 2 samples (2 pools of five swabs or 2 oral fluids) would be submitted for testing from each farm with >2000 pigs, and one sample (1 pool of five or 1 oral fluid) would be submitted from each farm with ≤2000 pigs.

3. Testing would be performed at laboratories in the USDA National Animal Health Laboratory Network (NAHLN) [58]. As of June 2025, the NAHLN included > 60 federal, state, and university-affiliated laboratories. To evaluate the accessibility of NAHLN laboratories with respect to producers, the distance from each swine farm (*n* = 66,637) to the nearest NAHLN laboratory was calculated using data from OpenStreetMap and the open-source routing machine method [59]. Among all farms (Figure 4), this distance ranged from 2.4 km (1.49 miles) to 887.7 km (551.6 miles) with a mean distance of 192.3 km (119.4 miles).

### 2.5. Phase 3 Results: Cost of Sampling and Testing

Sampling and shipping costs (Table 5) were based on 2024 U.S. market prices for specimen-specific sampling supplies (oral fluids, oral swabs, or blood swabs) and shipping services. The farm-level cost per round of sampling varied by specimen type: oralfluid sampling supplies totaled $3.91 (€3.65), oral swabs cost $2.88 (€2.69), and blood swabs cost $3.88 (€3.62). Shipping costs were calculated based on standard shipping rates for 2 types of packaging: a laboratory test tube mailer (20.3 × 15.2 × 12.7 cm, with a capacity of five 15-mL tubes) or an insulated cooler (17.7 × 12.7 × 3.8 cm). In compliance with federal regulations (49 CFR § 173.199), for shipping costs, we assumed appropriate packing supplies would be used, e.g., triple packaging and ice packs. Laboratory testing costs were assumed to be $8.00 USD (€7.80) for antibodies (ELISA) and $30.00 (€29.25) for nucleic acid (PCR) per sample.

**Figure 5:**
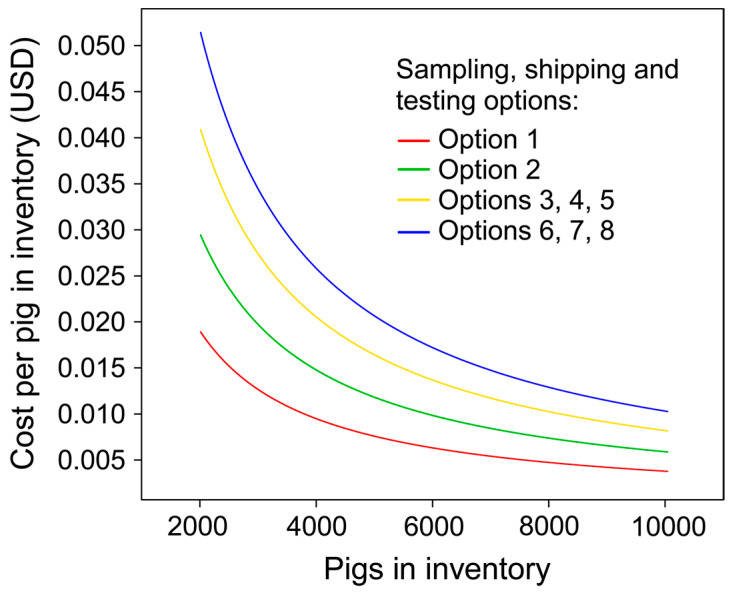
Cost per pig in inventory on farms with ≥2000 pigs based on sampling, shipping, and testing options: (1) oral fluid, laboratory tube mailer, and ELISA; (2) oral fluid, insulated cooler, and ELISA; (3) oral swab, laboratory tube mailer, and PCR; (4) blood swab, laboratory tube mailer, and PCR; (5) oral fluid, laboratory tube mailer, and PCR; (6) oral swab, insulated cooler, and PCR; (7) blood swab, insulated cooler, and PCR; and (8) oral fluid, insulated cooler, and PCR.

Based on the 8 sampling, shipping, and testing combinations defined in Table 6, the total cost per round of sampling on farms with ≤2000 pigs ranged from $27.97 (€26.12) to $71.04 (€66.33). On farms with >2000 pigs, the cost ranged from $37.84 (€35.33) to $102.90 (€96.09). In both farm size categories, differences in cost were primarily driven by producer choices regarding shipping (laboratory tube mailer vs. insulated cooler) and laboratory testing (ELISA vs. PCR). Figure 5 illustrates the effect of sampling, shipping, and testing choices on the cost per pig in inventory for farms with >2000 pigs.

## 3. Discussion

The structure of contemporary swine production and its production practices create the need for an active, effective surveillance system to facilitate the rapid detection, containment, and elimination of notifiable pathogens. At the global level, swine production has evolved toward fewer, larger farms [60]. In the U.S., for example, the number of swine farms declined from ~4.85 million farms housing ~60 million pigs [61] in 1920 to 66,637 farms housing ~71 million pigs in 2017 [45]. Aligned with these demographic changes, the U.S. swine industry implemented the practice of locating sow farms in one region and then transporting weaned pigs to finishing farms located elsewhere [51,52,62]. Thus, in 2024, 63,484,200 pigs were transported across state borders to farms in other states for feeding or breeding purposes [63]. In the U.S. and elsewhere, networks of larger, highly interconnected metapopulations have facilitated the introduction and rapid spread of pathogens via the movement of animals, people, and materials. Hence, following its introduction in 2013, PEDV spread across thirteen U.S. states (>200 herds) in less than two months [64].

In this study, we tested the hypothesis that collecting and testing diagnostic specimens from a few targeted pigs on each of many farms could effectively achieve the detection of a notifiable pathogen in a large geographic region. Disease spread simulations were based on the 2017 USDA Census of Agriculture data, i.e., 66,637 swine farms in the continental U.S. (8,080,470 km^2^). Modeling pathogen spread at a regional level is, at best, an approximation limited by the inherent complexity, variability, and stochasticity of transmission probabilities in a dynamic population in a large geographic region [65]. As Box (1976) explained, “Since all models are wrong, the scientist cannot obtain a ‘correct’ one by excessive elaboration [66].” For this reason, the ADSM simulations were designed to cover a variety of transmission possibilities. That is, rather than limiting the disease spread simulations to a single pathogen or fixed set of parameters, we used a range of transmission probabilities to cover a broad spectrum of potential spread scenarios.

In 2017, U.S. farms with <2000 pigs in their inventories represented 87% of all operations and held ~7% of the national herd, while farms with >2000 pigs in their inventories made up 13% of the total operations and held ~93% of the national herd [67]. Thus, sample size, frequency, and ease of collection are major considerations in participatory surveillance because smaller farms remain a significant proportion of the industry, and their inclusion in participatory surveillance is critical. The antemortem targeted sampling approach used in this surveillance design has proven to be an efficient approach to disease detection. For example, Williams et al. (2009) determined that random sampling would require five to seven times more samples than targeted sampling to achieve the same level of confidence [68]. Moreover, the use of antemortem samples would allow for subsequent resampling to confirm or rule out a non-negative result. More specifically, collecting diagnostic specimens from poor-doing pigs has been previously described for ASFV and CSFV surveillance [56,57,69]. Malladi et al. (2022) predicted that ASFV in finisher herds would be detected within ~2 weeks based on simulations of an active surveillance strategy targeting sick or dead pigs [69]. Similarly, EFSA et al. (2021) modeled targeted sampling for ASFV and reported that the collection of samples from at least five pigs (dead or with clinical signs) enabled detection with 95% confidence at 13 days post-introduction [56,57].

The diagnostic specimens described in this design, i.e., oral fluids, oral swabs, and blood swabs, were selected based on considerations of feasibility, practicality, and their demonstrated effectiveness for pathogen detection [70,71,72,73]. The use of these specimens has been described for the detection of most swine pathogens, including ASFV, CSFV, FMDV, influenza A virus, porcine circovirus 2, porcine deltacoronavirus, PEDV, porcine parvovirus, porcine reproductive and respiratory syndrome virus (PRRSV), and others [74]. A further advantage in surveillance is that aggregate samples, such as pen-based oral fluids, include diagnostic material from many individuals in the population, thereby increasing the likelihood of pathogen detection [55]. Recently, the WOAH described pen-based oral fluids as “ … a convenient, non-invasive means of sampling at a herd or pen level” in ASFV surveillance [72].

Diagnostic laboratory capacity is a limited resource that must be included in the planning process; for this reason, the expected testing volume resulting from a national implementation of participatory surveillance was calculated in this study. Overall, 20% producer participation (13,327 farms) resulted in 15,090 tests per round of sampling. It follows that 40% producer participation (26,655 farms) would result in 30,180 tests, 60% producer participation (39,982 farms) would result in 45,269 tests, 80% producer participation (53,310 farms) would result in 60,359 tests, and 100% producer participation (66,637 farms) would result in 75,499 tests. While significant, these numbers are well within the capacity of the >60 laboratories in the U.S. NAHLN network, even with 100% producer participation. For example, between 2020 and 2021, 33 NAHLN laboratories tested > 5.6 million samples to support the U.S. emergency response to the SARS-CoV-2 pandemic in addition to their routine diagnostic caseload [75].

## 4. Conclusions

The performance of active participatory surveillance in the continental U.S. was evaluated in terms of the probability of detecting a notifiable pathogen over time post-introduction as a function of farm-level sensitivity, farm-level prevalence, producer participation, and repeated sampling. Even at relatively low farm-level sensitivity (≥20%) and producer participation levels (≥40%), participatory surveillance achieved ≥ 90% probability of detection at 0.05% prevalence (with 33 infected among 66,637 farms). That is, the design proved to be highly sensitive at a cost of $0.03 to $0.07 USD (€0.02 to €0.06) per pig in inventory, depending on the sampling and testing choices. Integration of participatory surveillance with the NAHLN laboratory network is justified both based on the diagnostic expertise of these laboratories and by the fact that these laboratories uniformly use laboratory information management systems (LIMS) and electronic messaging systems that facilitate rapid reporting to animal health authorities and timely decision-making [76]. Thus, active participatory surveillance is adaptable for use with both endemic and exotic pathogens across diverse production systems and regions worldwide and could play an important role in limiting disruption of interstate and international commerce during disease outbreaks.

## Figures and Tables

**Table 1:** Transmission probability parameters set in the Animal Disease Spread Model (ADSM) software to simulate pathogen spread (Phase 1) in a population of 66,637 swine farms holding 71,555,218 pigs across a contiguous region of 8,080,470 km^2^.

Disease Spread Parameter	Parameter Definition and Value			
**1.** Index farm:	**1.** First positive farm in each simulation.			
**a.** Pig density per km^2^.	**a.** <1 pig, 1 to 10 pigs, 10 to 100 pigs, >100 pigs.			
**b.** Production type.	**b.** Breeder, feeder, breeder–feeder.			
**2.** Direct contact:	**2.** Probability of transmission via movement of infectious animals among farms.			
**a.** Distance for direct contact.	**a.** Min, 0.5 km; mode, 100 km; max, 1000 km (BETAPert distribution).			
**b.** Daily movement rate.	**b.** Average daily contact by farm through the shipment of animals:			
	**Destination**			
	**Source herd**	Breeder	Feeder ^1^	Packing plant ^2^
	Breeder–Feeder	NA	0.0204	0.0310
	Breeder	0.0014	0.0687	0.0310
	Feeder	NA	0.0348	0.0310
**c.** Probability of infecting a negative farm.	**c.** 0.2, 0.4, 0.6.			
**3.** Indirect contact:	**3.** Probability of transmission via contaminated personnel, fomites, etc.			
**a.** Distance for indirect contact.	**a.** Min, 0.5 km; mode, 100 km; max, 1000 km (BETAPert distribution).			
**b.** Daily indirect-contact rate.	**b.** Average daily indirect contact generated by each farm:			
	**Destination**			
	**Source herd**	Breeder–Feeder	Feeder ^1^	Packing plant ^2^
	Breeder–Feeder	NA	0.0001	0.0005
	Breeder	0.0001	0.0043	0.0013
	Feeder	0.0005	0.0015	0.0156
**c.** Probability of infecting a negative farm.	**c.** 0.05, 0.1, 0.15			
**4.** Local area spread:	**4.** Daily probability of spread to farms located ≤1 km from the infected farm.			
**a.** Probability of infecting a negative farm.	**a.** 0.001, 0.01, 0.1 (exponential drop off).			

^1^ Thakur et al., 2015 [53]. ^2^ Machado et al., 2021 [54].

**Table 2:** Total number of infected farms (day 180 post-introduction) by disease spread category (see Figure 3) as a function of disease spread parameters.

Pig Density ^3^	Production Type ^1,3^	Local Area Spread ^1,2,3^	Direct Contact ^1,2,3^								
			0.2			0.4			0.6		
			Indirect Contact								
			0.05	0.1	0.15	0.05	0.1	0.15	0.05	0.1	0.15
<1 pig/km^2^	BF	0.001	6	6	7	25	27	31	79	90	103
	Breeder		14	15	16	72	83	93	226	251	266
	Feeder		8	10	11	33	39	45	101	117	135
1 to 10 pigs/km^2^	BF		6	6	7	26	29	34	88	100	115
	Breeder		15	17	23	93	107	121	324	463	487
	Feeder		9	11	12	39	45	53	124	145	166
10 to 100 pigs/km^2^	BF		6	6	7	28	31	35	93	108	123
	Breeder		20	22	23	100	116	132	340	463	505
	Feeder		9	11	13	42	49	57	134	156	181
>100 pigs/km^2^	BF		7	7	8	35	40	45	115	132	150
	Breeder		21	23	26	108	123	140	351	477	498
	Feeder		10	12	13	48	56	65	151	176	186
<1 pig/km^2^	BF	0.01	6	7	7	31	35	40	103	119	135
	Breeder		15	15	24	86	99	111	256	543	577
	Feeder		9	10	12	41	48	56	132	152	166
1 to 10 pigs/km^2^	BF		7	7	8	33	38	42	115	134	151
	Breeder		16	23	26	113	130	147	487	522	569
	Feeder		11	12	14	49	57	66	161	189	216
10 to 100 pigs/km^2^	BF		7	8	9	37	42	48	130	149	168
	Breeder		23	25	28	128	146	166	497	546	618
	Feeder		12	14	15	55	65	74	182	211	244
>100 pigs/km^2^	BF		9	10	11	50	57	64	148	168	192
	Breeder		26	29	33	143	163	184	509	582	652
	Feeder		13	14	16	67	78	90	200	232	265
<1 pig/km^2^	BF	0.1	12	12	13	114	129	134	758	758	827
	Breeder		39	44	50	233	265	557	1217	1370	1512
	Feeder		13	14	35	138	147	167	791	849	923
1 to 10 pigs/km^2^	BF		14	14	32	140	156	174	718	797	882
	Breeder		55	62	72	554	604	598	1786	2018	2197
	Feeder		33	39	44	195	210	234	904	977	1089
10 to 100 pigs/km^2^	BF		42	47	48	209	232	245	969	1038	1149
	Breeder		82	93	106	669	733	783	2313	2581	2812
	Feeder		51	59	67	271	561	562	1210	1344	1493
>100 pigs/km^2^	BF		139	156	173	764	852	927	2023	2220	2414
	Breeder		195	217	225	1067	1182	1304	3433	3736	4029
	Feeder		155	174	192	815	871	971	2310	2534	2773

^1^ Significant spread parameter at α = 0.01 (chi-squared test) for slow spread (teal). ^2^ Significant spread parameter at α = 0.01 (chi-squared test) for moderate spread (yellow). ^3^ Significant spread parameter at α = 0.01 (chi-squared test) for fast spread (orange).

**Table 3:** Probability (%) of detecting ≥ 1 positive farm at specific farm-level prevalences as a function of producer participation and farm-level sensitivity ^1^.

Prevalence ^2^	Farm-Level Sensitivity ^3^	Producer Participation				
		20%	40%	60%	80%	100%
0.010% (7 farms)	10%	0.13	0.25	0.35	0.44	0.52
	20%	0.25	0.44	0.59	0.70	0.79
	30%	0.35	0.59	0.75	0.85	0.92
	40%	0.44	0.70	0.85	0.93	0.97
	50%	0.52	0.79	0.92	0.97	0.99
0.050% (33 farms)	10%	0.49	0.74	0.87	0.94	0.97
	20%	0.74	0.94	0.98	0.99	0.99
	30%	0.87	0.98	0.99	1.00	1.00
	40%	0.94	0.99	1.00	1.00	1.00
	50%	0.97	0.99	1.00	1.00	1.00
0.102% (68 farms)	10%	0.74	0.93	0.98	0.99	0.99
	20%	0.93	0.99	1.00	1.00	1.00
	30%	0.98	1.00	1.00	1.00	1.00
	40%	0.99	1.00	1.00	1.00	1.00
	50%	0.99	1.00	1.00	1.00	1.00
0.200% (133 farms)	10%	0.93	0.99	1.00	1.00	1.00
	20%	0.99	1.00	1.00	1.00	1.00
	30%	1.00	1.00	1.00	1.00	1.00
	40%	1.00	1.00	1.00	1.00	1.00
	50%	1.00	1.00	1.00	1.00	1.00

^1^ Probability of detection calculated as the weighted average detection probability of all simulation results matching the selected prevalences for each combination of producer participation and farm-level sensitivity (R v.4.3.3). Values > 99% are highlighted in blue. ^2^ Farm-level prevalence in a population of 66,637 swine farms holding 71,555,218 pigs within an area of 8,080,470 km^2^. ^3^ Farm-level sensitivity = probability of a positive test for samples coming from a true-positive farm.

**Table 4:** Probability (%) of detecting ≥1 positive farm: Comparison of point-in-time (*P*) ^2^ vs. repeated sampling (*Pa*) ^3^ at 14-day intervals in detection probability as a function of farm-level prevalence by day post-introduction (DPI), producer participation, and farm-level sensitivity ^4^.

		**20% Producer Participation** **(13,327 farms)**					**40% Producer Participation** **(26,654 farms)**				
DPI (prevalence)		*Farm-level sensitivity* ^1^					*Farm-level sensitivity*				
	***P* ^2^** **/*Pa* ^3^**	*0.1*	*0.2*	*0.3*	*0.4*	*0.5*	*0.1*	*0.2*	*0.3*	*0.4*	*0.5*
14 (0.015%)	***P***	0.19	0.36	0.51	0.64	0.75	0.34	0.59	0.76	0.87	0.94
28 (0.026%)	***P***	0.31	0.54	0.71	0.83	0.91	0.52	0.79	0.92	0.97	0.99
	***Pa***	0.44	0.70	0.86	0.94	0.98	0.68	0.91	0.98	1.00	1.00
42 (0.043%)	***P***	0.45	0.72	0.87	0.95	0.98	0.70	0.92	0.98	1.00	1.00
	***Pa***	0.69	0.92	0.98	1.00	1.00	0.91	0.99	1.00	1.00	1.00
56 (0.073%)	***P***	0.64	0.89	0.97	0.99	1.00	0.87	0.99	1.00	1.00	1.00
	***Pa***	0.89	0.99	1.00	1.00	1.00	0.99	1.00	1.00	1.00	1.00
		**60% Producer Participation** **(39,982 farms)**					**80% Producer Participation** **(53,310 farms)**				
DPI (prevalence)		*Farm-level sensitivity*					*Farm-level sensitivity*				
	***P/Pa***	*0.1*	*0.2*	*0.3*	*0.4*	*0.5*	*0.1*	*0.2*	*0.3*	*0.4*	*0.5*
14 (0.015%)	***P***	0.47	0.74	0.88	0.95	0.98	0.57	0.83	0.94	0.98	1.00
28 (0.026%)	***P***	0.67	0.90	0.98	1.00	1.00	0.77	0.95	0.99	1.00	1.00
	***Pa***	0.82	0.97	1.00	1.00	1.00	0.9	0.99	1.00	1.00	1.00
42 (0.043%)	***P***	0.84	0.98	1.00	1.00	1.00	0.91	0.99	1.00	1.00	1.00
	***Pa***	0.97	1.00	1.00	1.00	1.00	0.99	1.00	1.00	1.00	1.00
56 (0.073%)	***P***	0.95	1.00	1.00	1.00	1.00	0.98	1.00	1.00	1.00	1.00
	***Pa***	1.00	1.00	1.00	1.00	1.00	1.00	1.00	1.00	1.00	1.00
		**100% Producer Participation** **(66,637 farms)**									
DPI (prevalence)		*Farm-level sensitivity*									
	***P/Pa***	*0.1*	*0.2*	*0.3*	*0.4*	*0.5*					
14 (0.015%)	***P***	0.65	0.89	0.97	0.99	1.00					
28 (0.026%)	***P***	0.84	0.98	1.00	1.00	1.00					
	***Pa***	0.94	1.00	1.00	1.00	1.00					
42 (0.043%)	***P***	0.95	1.00	1.00	1.00	1.00					
	***Pa***	1.00	1.00	1.00	1.00	1.00					
56 (0.073%)	***P***	0.99	1.00	1.00	1.00	1.00					
	***Pa***	1.00	1.00	1.00	1.00	1.00					

^1^ Probability of a positive test for samples coming from a true-positive farm. ^2^ Probability of detecting ≥ 1 positive farm for one-point-in-time sampling: *P* = 1 − (1 − *p*)*^n^*. ^3^ Aggregated probability of detection for repeated sampling: *Pa =* [1 − (1 − *P*_14_) × (1 − *P*_28_) ×… (1 − *P_DPI_*)]. ^4^ Values >99% are highlighted in blue.

**Table 5:** Estimated cost of sampling and shipping supplies per farm (>2000 pigs).

	Cost/Item ^1^	No. Items	Cost/Farm (USD)	Cost/Farm (EUR) ^2^
1. Sample collection				
A. *Oral fluid*				
Disposable gloves	$0.09	2 (1 pair)	$0.17	€0.16
Cotton rope (1/2”)	$0.38/ft	7 feet	$2.76	€2.58
Resealable plastic bag	$0.14	2	$0.29	€0.27
Plastic tube (5 mL)	$0.35	2	$0.69	€0.64
			$3.91	€3.65
B. *Oral swab* ^3^				
Disposable gloves	$0.09	4 (2 pairs)	$0.34	€0.32
Polyester swab	$0.08	10	$0.85	€0.79
Plastic tube (15 mL)	$0.62	2	$1.24	€1.16
Transport medium ^4^	$0.05/mL	10 mL	$0.46	€0.43
			$2.88	€2.69
C. *Blood swab* ^3^				
Disposable gloves	$0.09	4 (2 pairs)	$0.34	€0.32
Polyester swab	$0.08	10	$0.85	€0.79
20-gauge needle	$0.10	10	$1.00	€0.93
Plastic tube (15 mL)	$0.62	2	$1.24	€1.16
Transport medium ^4^	$0.05	10 mL	$0.46	€0.43
			$3.88	€3.62
2. Shipping to laboratory				
A. *Laboratory tube mailer*				
Tube mailer	$2.14	1	$2.14	€2.00
Ice packs (3 oz)	$0.38	1	$0.38	€0.35
Shipping fee	$15.41	-	$15.41	€14.39
			$17.93	€16.74
B. *Insulated cooler*				
Insulated foam cooler	$8.33	1	$8.33	€7.78
Ice packs (24 oz)	$1.42	1	$1.42	€1.33
Whirl bag	$0.34	1	$0.34	€0.32
Shipping fee	$28.91	-	$28.91	€26.99
			$39.00	€36.41

^1^ Prices are based on U.S. distributor rates as of 2024. ^2^ EUR (€) 1.00 = USD ($) 1.0710 (https://www.federalreserve.gov/releases/h10/current/, accessed on 8 November 2024). ^3^ Samples were assumed to be pooled (5 pigs per pool) prior to shipment to avoid sample-processing charges at the laboratory. ^4^ Phosphate-buffered saline.

**Table 6:** Cost of one round of sampling and testing for small (≤2000 pigs) and large (>2000 pigs) farms based on 8 sample, shipping, and testing options.

				Cost per Farm	
Option	Specimen	Shipping	Analyte	≤2000 Pigs	>2000 Pigs
1	Oral fluid	Lab tube mailer	ELISA ^1^	$27.97 (€26.12)	$37.84 (€35.33)
2	Oral fluid	Insulated cooler	ELISA	$49.04 (€45.79)	$58.91 (€55.00)
3	Oral swab	Lab tube mailer	PCR ^2^	$49.37 (€46.10)	$80.81 (€75.45)
4	Blood swab	Lab tube mailer	PCR	$49.87 (€46.56)	$81.81 (€76.39)
5	Oral fluid	Lab tube mailer	PCR	$49.97 (€46.66)	$81.84 (€76.41)
6	Oral swab	Insulated cooler	PCR	$70.44 (€65.77)	$101.8 (€95.13)
7	Blood swab	Insulated cooler	PCR	$70.94 (€66.24)	$102.8 (€96.06)
8	Oral fluid	Insulated cooler	PCR	$71.04 (€66.33)	$102.90 (€96.09)

^1^ Antibody ELISA ($8.00; €7.80). ^2^ PCR ($30.00; €29.25).

## Data Availability

The original contributions presented in this study are included in the article. Further inquiries can be directed to the corresponding author.
